# Association of temperament with genetic polymorphisms in *SOD1*, *GSTM1* and *GSTT1* genes

**DOI:** 10.22099/mbrc.2020.38820.1565

**Published:** 2021-03

**Authors:** Zahra Zendehboodi, Zahra Saberikia

**Affiliations:** Department of Biology, College of Sciences, Shiraz University, Shiraz 71467-13565, Iran

**Keywords:** Temperament, GSTT1, GSTM1, SOD1, polymorphism

## Abstract

Due to its accessibility, efficacy, and affordability, traditional medicine (TM) is the main source of health services for many people in the world. Nevertheless, in spite of its benefits, there are still many issues about the principles of TM which demand further declaration. One of the essential principles of Iranian traditional medicine (ITM) is temperament (mizaj), which efficiently applied in diagnosis and therapy of illnesses. In this study we aimed to explore the association of *GSTM1*/*T1*, and *SOD1* 50 bp Ins/Del polymorphisms with combined groups of temperament. The study was conducted in 217 healthy males from Fars province, southern Iran. The self-reported mizaj questionnaire was applied to identify the participants**’ **temperament. Then individuals with temperate, warm/moist, and warm/dry temperament were entered in the study. To determine the genotype of *GSTM1, GSTT1, *and *SOD1*, the polymerase chain reaction (PCR)-based method was performed. As the results of χ^2 ^analysis showed, the frequency of *GSTT1*,* GSTM1*, and *SOD1 *polymorphisms in temperate group was not significantly differ from that in each of warm/moist and warm/dry groups. Further research with larger samples are suggested to clarify the association between temperament and biomolecular features.

## INTRODUCTION

One of the schools of traditional medicine with long history is Iranian traditional medicine (ITM). In addition to disease prevention and treatment, ITM attempts to provide the instructions for a person to maintain the body function at the best state of health [1]. One of the essential concepts of this system is temperament, which efficiently applied in diagnosis and therapy of illnesses. Temperament is considered as a new homogenous quality arising from the interaction of four elements of earth, fire, air, and water with different natures (cold, hot, dry, and wet) in the human body. Since the potency of warmness and wetness correlates with specific body and psychic features, large numbers of temperament may be assumed; however based on warmness and wetness intensity, ITM classify the temperaments into 9 major groups: one temperate group; four simple groups (cold, warm, dry, and moist); and four combined groups (warm/dry, warm/moist, cold/dry, and cold/moist). The disturbance in body temperament is believed to be the reason of occurrence of disease [2, 3]. 

The glutathione transferases (GSTs) are multifunctional enzymes in both eukaryotes and prokaryotes. They plays a critical role in cellular detoxification against xenobiotic, carcinogens, and oxidative stress. Three main groups of GSTs in eukaryotes are cytosolic, mitochondrial and microsomal GSTs. Several classes of cytosolic GSTs in human were identified including zeta, alpha, mu, theta, pi, omega and sigma. The GST genes coding for *GSTM1* (class mu member; OMIM: 138350) and *GSTT1* (class theta member; OMIM: 600436) have been shown to hold the gene deletion polymorphism. Homozygosity for the deleted gene resulted in *GSTM1*/*GSTT1* null genotype. The association of deletion polymorphisms of *GSTT1* and *GSTM1* with a number of cancers and diseases such as uterine leiomyoma, breast cancer, hypertension, oral leukoplakia, and susceptibility to drug dependence was investigated [4-6].

Superoxide dismutases (SOD) are most powerful antioxidant enzymes in the cell and considered as a components of first line of defense against reactive oxygen species (ROS). Different genes encode diverse isozymes of SOD. The superoxide dismutase-1 (*SOD1*, OMIM: 147450) gene encodes Cu/Zn-SOD which promotes the superoxide anions dismutation to molecular oxygen and hydrogen peroxide [7]. Reactive oxygen species (ROS) are extremely reactive agents that are normally produced during the body’s metabolic processes. At physiological low levels, ROS can be operated in regulatory mechanism and cell signaling while in surfeit, they give rise to oxidative change of molecules such as lipid, protein or DNA, resulting in cellular damage or promotion of cell death [8]. Among detected genetic polymorphisms of *SOD1*, there is a polymorphism of 50 bp insertion/deletion (Ins/Del) in the promoter area of this gene [9], which has been shown to be associated with reduced mRNA expression of *SOD1* [10]. Association of *SOD1* gene polymorphisms with the risk of age onset of some mental disorders, cardiovascular disease, and gastric and colorectal cancers has been reported [11-16]. 

Our previous study showed that biomarkers of apoptosis (condensed chromatin, pyknosis and karyorrhexis) and necrosis (karyolysis) [17] were not evenly distributed in all temperament groups. Also the frequency of nuclear bud, a DNA damage marker, in warm/moist temperament group significantly decreased compared to the moist group [18]. Apoptosis, necrosis, and nuclear bud are considered to be induced by oxidative stress [19-21]. Moreover, we examined the association of the polymorphisms in the antioxidant enzyme genes including *GSTM1/T1*, and *SOD1* Ins/Del with simple groups of temperament and found out an increased frequency of *GSTT1*-null genotype, although not at substantial levels, in warm groups compared to the individuals who are temperate for warmness [22, 23]. Based on these evidence we hypothesized that the temperament features may be affected by antioxidant system. Therefore, in the present study we focus on the association of *GSTM1*/*T1* and *SOD1* Ins/Del polymorphisms with combined groups of temperament.

## MATERIALS AND METHODS

**Study population: **The study was conducted in 217 healthy males referred for blood donation to Shiraz Blood Transfusion Organization, Fars province, southern Iran. The age of participants was between 20-40 years. The self-reported mizaj questionnaire constructed by Mojahedi et al for healthy individuals [24] was used to identify the participants**’ **temperament. After mizaj determination, individuals with temperate or any of 4 combined mizaj groups (warm/dry, warm/moist, cold/dry, cold/moist) were selected. Because of low number of individuals with cold/moist and cold/dry temperament, these groups were not included in the study. This study was approved by Shiraz University ethics committee and informed consent was provided by all individuals.

**DNA extraction and genotyping: **Blood specimens derived from donors were preserved at -20°C until use. Extraction of genomic DNA from thawed blood samples was performed by standard procedure [25]. To genotype *GSTM1* and *GSTT1 *multiplex polymerase chain reaction (PCR) was done. The PCR procedure was performed using the following *GSTT1* primers: F, 5'-TTC CTT ACT GGT CCT CAC ATC TC-3’ and R, 5'-TCA CCG GAT CAT GGC CAG CA-3’; *GSTM1* primers: F, 5'-GAA CTC CCT GAA AAG CTA AAG C-3’ and R, 5'-GTT GGG CTC AAA TAT ACG GTG G-3’; and β-globin primers: F, 5'-CAA CTT CAT CCA CGT TCA CC-3’ and R, 5'-GAA GAG CCA AGG ACA GGT AC-3’ [26, 27]. β-globin gene was utilized as an internal control. The reaction mixture was subjected to the following condition: initial denaturation for 5 min at 94°C; 32 cycles of denaturation for 1 min at 94°C, primer annealing at 65.5°C for 40 secs, and primer extension at 72°C for 1 min; and a final extension at 72°C for 5 min. The PCR outcomes of *GSTT1*, *GSTM1*, and β-globin genes were 459, 219, and 268 bp fragments, respectively. Individuals with homozygous null genotypes of *GSTM1 *and *GSTT1* were distinguished by the absence of amplified product. Successful amplification by specific primers of β-globin indicated the proper operation of the PCR procedure. The genotypes of 55 individuals in temperate group were provided from our previous study [22]. The PCR primers and conditions for genotyping of *SOD1* were the same as that previously reported [11]. The PCR outcomes of *SOD1* gene were 297 and 247 bp fragments. The genotypes of 58 individuals in temperate group were provided from our previous study [23]. 

**Statistical analysis:** The χ^2 ^test was applied to asses if the frequency of *GSTT1*, *GSTM1*, and *SOD1 *genotypes in each combined mizaj group was differ from that in the temperate group. ANOVA was used to determine the difference of age mean between the study groups. The Hardy-Weinberg equilibrium (HWE) was examined using the online Court lab - HW calculator- imp ortant.xls software. The SPSS version 22 was used for statistical analysis. The result were presented as number of allele or genotype, and significance was accepted at P<0.05.

## RESULTS

The present study included 76, 47, and 94 individuals with warm/moist, warm/dry, and temperate temperament respectively. The age mean of the warm/moist, warm/dry, and temperate groups were 30.7±5.3, 31.6±4.9, and 32.4±5.0, respectively. The difference between the means of age in different temperament groups was not significant (F=2.42; df=2, 214; P>0.05). 

Totally, for *SOD1* Ins/Del polymorphism, 6, 49, and 161 individuals hold Del/Del, Ins/Del and Ins/Ins genotypes respectively (Table 1). The overall allelic frequencies of *SOD1* insertion/deletion polymorphism were 86 and 14% for Ins and Del alleles respectively and according to the HWE the expected genotypic frequencies did not significantly differ from the observed genotypic frequencies (χ^2^=0.90, df=1, P=0.342). 

**Table 1:** Association between temperament and polymorphisms of *SOD1*, *GSTM1*, and *GSTT1*

**Genotype**	**Temperament**				**df**	**P**
	**Warm/Dry**	**Warm/Moist**	**Temperate**			
***SOD1***						
**Ins/Ins**	33	56	72	0.674^*^	1	0.714
**Ins/Del**	13	16	20			
**Del/Del**	1	3	2			
**Del/-**	14	19	22			
						
***GSTT1***						
**Active**	36	60	76	0.352	1	0.839
**Null**	11	16	18			
						
***GSTM1***						
**Active**	19	32	46	1.237	1	0.539
**Null**	28	44	48			

*The analysis was performed between individuals with Ins/Ins and Del/- genotypes.

Because the multiplex PCR assay we used for *GSTT1* and *GSTM1* genotyping could not discriminate the homozygous form the heterozygous genotype, we could not performed the HWE examination in the sample for these two genotypes. Due to low frequency of the Del allele of *SOD1*, the genotypes of Del/Del and Ins/Del were pooled and considered as the Del/- genotype for statistical analysis. One of the samples was not successfully genotyped for *SOD1*. The distribution pattern of *GSTT1*, *GSTM1*, and* SOD1 *genotypes in the study groups were examined using χ^2 ^test. As the results showed (Table 1), the frequency of *GSTT1*, *GSTM1*, and* SOD1 *polymorphisms was not significantly different between the study groups.

## DISCUSSION

In the opinion of ITM, mizaj acts as an essential guideline in lifestyle orders, diagnosis, and treatment of diseases [2]. On the other hand, for the maintenance of health and prevention of diseases it is necessary to keep temperament in its balanced condition. On that account, rectifying of mizaj is one of the main method of diseases treatment in ITM health care system [3]. Due to its accessibility, efficacy, affordability, and culturally acceptability by people, TM is the main source of health services for many people in the world [28]. Nevertheless, in spite of its benefits, there are still many issues about the principles of TM which demand further declaration. Meanwhile, a growing body of evidence have support the connection of temperament with individual genetic background. It is found that genes correlated with hot ZHENG-related disorders are components of the pathway of cytokine–cytokine receptor interaction, whereas genes correlated to both the hot and cold -related disorders are connected to the pathway of the neuroactive ligand-receptor interaction [29]. Investigating the pattern of gene expression in cold syndrome patients showed that genes associated to cold syndrome involve in energy metabolism [30]. The gene expression profiles examination in CD4+ T cells from rheumatoid arthritis patients carrying hot and cold pattern indicated that some genes differentially expressed between this two groups and patients carrying hot pattern showed an increased expression of the genes linked to fatty acid metabolism, pathways of small G protein signaling, and T cell proliferation [31]. Proteome and network analysis in normal individuals with hot-wet, and cold-dry temperament evidenced that some of mitochondrial proteins are related to or differentially overexpressed in specified group [32]. This data when put together, reinforce a biomolecular basis for the temperament.

Previously the association between simple mizajs and polymorphisms in each of antioxidant enzymes of *SOD1* and *GSTT1* and *GSTM1* was investigated and the warm temperament was fond to be possibly correlated with *GSTT1* genotype [22-23]. In this study the association between the same gene polymorphisms and combined groups of temperament was assessed. Some of the features of individuals with warm/moist temperament are having low fat, black and straight hair, red and with skin; being susceptible to infectious diseases, temperate in psychic behaviors, etc. Individuals with warm/dry temperament are distinguished by abundant and black hair, warm, hard, and brunet skin, strong digestive system, braveness, etc. [3]. Individuals with temperate temperament are in rather medium state for mizaj identification indices such as condition of touch (warm/cold), hair (curly/straight), sleep and wakefulness, physical and psychic functions, etc. [2] As the result of this study showed, no any difference in the frequency of *GSTM1*/*T1 *and *SOD1* polymorphisms between temperate group and each of warm/moist and warm/dry ones was detected. However, it should be mentioned that because of missing of two other temperament groups (cold/moist and cold/dry), we might have lost the significant findings as we could not examine the effect of these polymorphisms on the studied temperament groups compared to the other combined ones.This is the first study of its kind and further research with larger sample are needed to clarify the association between temperament and biomolecular features.
